# Magneto-Optical properties of noble-metal nanostructures: functional nanomaterials for bio sensing

**DOI:** 10.1038/s41598-018-30862-3

**Published:** 2018-08-23

**Authors:** Maria Grazia Manera, Adriano Colombelli, Antonietta Taurino, Antonio Garcia Martin, Roberto Rella

**Affiliations:** 1 Istituto per la Microelettronica e i Microsistemi IMM sezione di Lecce, Via per Arnesano, 73100 Lecce, Italy; 2 Instituto de Micro y Nanotecnología IMN-CNM, CSIC, CEI UAM+CSIC, Isaac Newton 8, Tres Cantos, Madrid 28760, Spain, Madrid, Spain

## Abstract

Metallic nanostructures supporting Localized Surface Plasmon Resonances (LSPR) are characterized by their unique ability to control and manipulate light at the nanoscale. Noble metal nanostructures, such as gold nanostructures, are demonstrating to exhibit magneto-optic activity in the presence of modulated magnetic field of low intensity in transversal configuration (T-MOKE). Validation of experimental findings was achieved by numerical simulations based on Finite Element Method (FEM) techniques. The developed numerical models allowed studying the combination of the T-MOKE effect with the localized surface plasmon resonance of metal nanoparticles. Numerical optical and magneto-optical spectra provided a deep insight on the physical aspects behind the magneto-optical activity of metal nanostructures strictly related to direction of oscillations electrical dipoles generated in resonance conditions. Additionally the MO signal was characterized as a transducing signal for refractive index sensing in liquid conditions. The outcome is an increase in the limit of detection of magneto optical transducer with respect to traditional plasmonic sensors. A new strategy for magneto-plasmonic sensing based on the use of glass supported -Au nanostructures based on their MO properties has put forward.

## Introduction

Nanoplasmonics is a branch of optical condensed matter science devoted to the study of optical phenomena in nanostructured metal systems. A remarkable property of such systems is their ability to concentrate the optical energy on the nanoscale due to the excitation of electromagnetic (E.M.) modes, called surface plasmons (SPs)^1,2^. They are strongly localized at the interfaces between two media with permittivity of opposite sign, such as the interface between a dielectric and a metal. For some particular geometries, when incident radiation couples to such SP modes, clear signatures in optical response of the system can be observed. Localized surface plasmon (LSP) modes occur in isolated, particle-like systems, and are responsible for the peaks observed in the absorption spectra of metallic nanoparticles. The position of the absorption maxima depends markedly on the metal and on the refractive index of the environment surrounding the nanoparticles. This is why they are often used as nanoantennas for single molecular recognition and for gas sensing applications^3^. In all these contexts the scientific interest is focused primarily on chip-based assay consisting of glass supported-metal nanoparticles which ensures rapid, cost-effective, and high-throughput measurements while simultaneously preventing aggregation, and hence convolution of the sensor response with plasmon coupling effects. Generally, in these cases, the incident light is coupled to the SPs modes of the metallic nanoparticles in the Total Internal Reflection (TIR) mode (also known as Kretschmann configuration).

However, sensing functionalities are not the unique properties of metal nanostructures. The excitation of the SPs modes may also affect their Magneto-Optical (MO) response. MO activity was demonstrated by using a meta-surface of nickel nanoantennas in different MO Kerr Effect geometries, i.e. longitudinal (L-MOKE), polar (P-MOKE) and transversal (T-MOKE)^4^. This property was recently used to demonstrate the MO response in nanostructures realized by noble metals and ferromagnets^5–7^. TMOKE effects were also analysed by considering a two-dimensional square array of gold nanoparticles embedded into a thin magnetic garnet films: an enhanced T-MOKE effect due to the excitation of quasi-waveguiding mode, with light concentrated inside a magnetic field, was recorded^8^. These studies demonstrate in a fascinating way the possibility of modulating the optical response by means of an external agent, namely a magnetic field. This research field has attracted growing scientific interest recently, owing to the great possibility of add active functionalities to metal metasurfaces. The term active refers to the ability of modulate a physical property by the presence of an external agent: in this case, an external magnetic field is used to modulate the MO properties of the nanoantennas upon a proper engineering of the metasurface design. Great possibility of achieving innovations in sensing, in light guiding devices and in optoelectronics are then opened.

The effect of plasmon excitation on the MO response in nanostructures made only of noble metal has not been studied but in a couple of works, both related to MO effects in gold nanoparticles. In the first, Sepulveda *et al*., demonstrated this effect by studying the MO response of nanodisks deposited onto a glass substrate in the P-MOKE configuration^5^. In the second, Pineider *et al*., demonstrated the existence magneto-plasmonic modes in gold nanoparticles dispersed in liquid solvents by magnetic circular dichroism (MCD) measurements^9^. In this last case the authors also attempted to use the effect for refractometric sensing by changing the refractive index of the solution. To the best of our knowledge, no experimental results are reported on T-MOKE characterization of glass supported -Au nanostructures, in particular when excited in Kretschmann configuration. This choice presents the practical advantage to pursuit a better signal-to-noise ratio, an essential requirements for high resolution sensing signals. Moreover, it is ideal for real time sensing applications, avoiding troubling interferences of light beam with analytes and buffer water solutions.

In this work, our intention is to try closing this scientific gap by analysing the TMOKE behaviour of Au nanostructures prepared by physical synthesis onto glass substrates. The presence of a significant MO effect on noble metal nanostructures is here demonstrated theoretically and experimentally, showing a clear enhancement of the TMOKE signal when LSP modes are excited upon illumination of the particles with p-polarized light. Finally, as a proof of concept, the potential application as a new transducing sensor signal of the recorded amplified MO signal is investigated. A noticeable improvement with respect to the sensing performance of the bare plasmonic signal is obtained, opening the perspective of sensitive detection of different surface biomolecular interactions using MO techniques and with well-known fabrication and functionalization protocols.

## Results and Discussion

### Morphological characterization

Let us now report on the characteristics of the samples. On the morphological side, Fig. S1 presents a high resolution Scanning Electron Microscopy (SEM) image of a typical “as deposited” e-beam evaporation sample, depicting a non-homogeneous distribution of gold clusters.

The unannealed islands present a rather complex shape, reflecting concurrent accumulation of Au on the substrate surface and coalescence of primary nucleated islands during evaporation.

**Figure 1:**
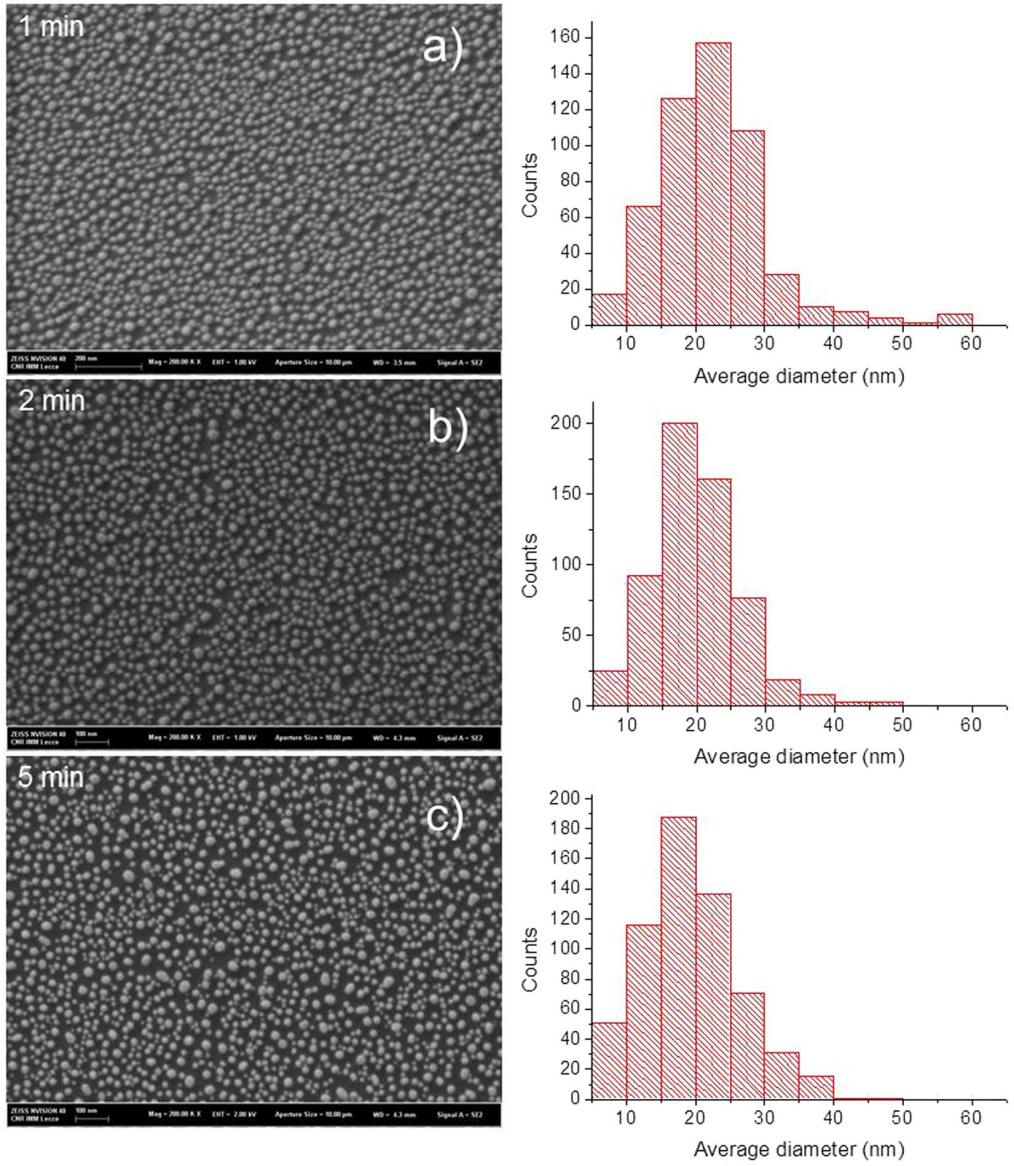
Morphology of the nanostructured gold nanoparticles obtained by SEM after a rapid thermal annealing treatment during (**a**) 1 minute (**b**) 2 minutes (**c**) 5 minutes. Statistical histogram of the nanostructures are reported on the right side.

The effect of the annealing process after cycles of 1 minute (b), 2 minutes (c) and 5 minutes (d), is presented in Fig. 1a–c. The figures present high-resolution SEM images of the samples together with histograms of the nanoparticle maximum diameter (since nanoparticles are not perfectly circular, a maximum and minimum diameter are considered to derive information about the nanoparticle size and shape). The sequence of images evidences the morphological evolution of the Au nanoparticles coverage due to the increase of the annealing time: after 1 minute, well developed nanoparticles form on the glass substrate and, as the annealing process proceeds, neighbouring nanoparticles tend to coalesce whereas isolated nanoparticles shrink, resulting in an average reduction of their diameter and in a likely increase of their height. These observations are supported by the results of the statistical analyses, reported in Table 1. As it can be observed, the particle sphericity increases with the annealing time, whereas the maximum diameter and the coverage percentage decrease.

**Table 1:** Statistical data related to the dimension of the nanoparticles and distribution.

Thermal annealing time	Maximum diameter (nm)	Sphericity	First neighbour distance (nm)	Coverage percentage
1 minute	(29 ± 9)	(0.62 ± 0.25)	(23 ± 5)	37
2 minutes	(21 ± 7)	(0.70 ± 0.20)	(23 ± 5)	31
5 minutes	(21 ± 8)	(0.79 ± 0.20)	(24 ± 6)	30

In general, rapid thermal annealing provides a sort of reshaping of the Au islands driven by capillary forces, coalescence and Ostwald ripening. As consequence, the initial distribution of inhomogeneous close islands comes into a distribution of quasi-spherical nanoparticles, characterized by separation distances of few nanometers.

### Optical characterization

**Figure 2:**
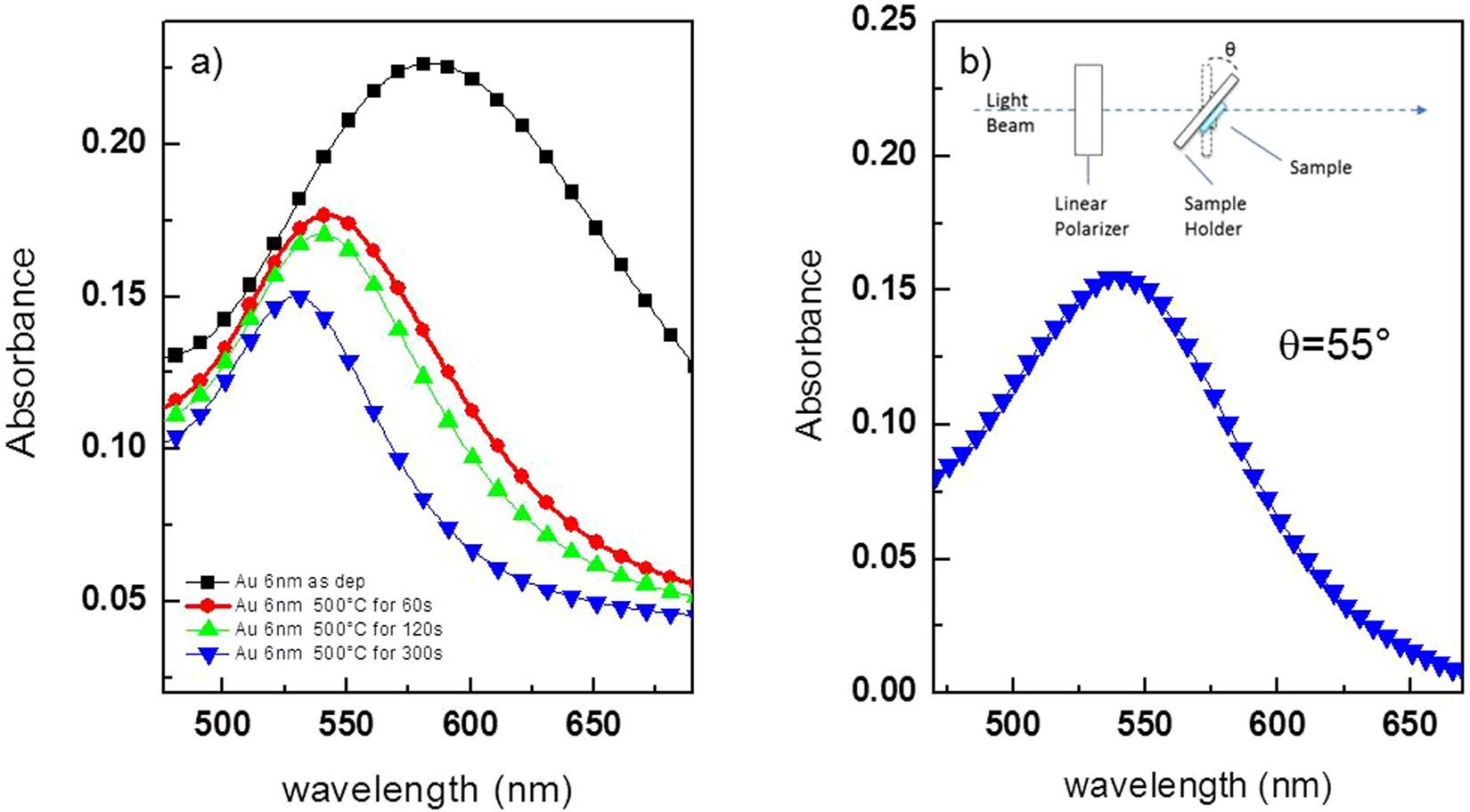
(**a**) Optical absorption spectra in the UV-VIS spectral range of a nanostructured Au samples before and after rapid thermal annealing treatments recorded at normal incidence. (**b**) Absorption spectrum of the sample annealed for 300 s recorded at the sample incidence angle chosen for the TMOKE characterization in Kretshmann configuration.

The significant changes experienced in the nanoparticle morphology due to thermal annealing have their echo in the corresponding optical absorption spectra. It is known that when metal NPs are brought into close proximity, strong electromagnetic coupling effects occur generating a great enhancement of their absorption spectra^10^. Therefore, broad and intense absorption spectra are expected for close-packed metal nanostructures, while narrowed and less-intense peaks are expended at spacing out the metasurfaces. Evolution of the optical spectra upon thermal annealing time is in agreement with the expected optical properties as shown in Fig. 2a. The unannealed islands, whose shape can be approximated as a lying prolate ellipsoid, showed a broad and intense SP band in the visible spectral range. From the optical point of view, annealing induces a blue shift in the spectrum and a decrease in the optical density accompanied by some band narrowing. Such effects became more evident as the annealing time increases, underling the close relation between morphology and optical properties^11^. Increase in the inter-island spacing with the annealing time, together with increase in the sphericity of nanostructures (Table 1) is reflecting in decoupling of SPs bands and progressive narrowing of the bandwidth. This latter aspect also pointed out that the resonance energy for plasmon is highly concentrated at the peak wavelength, in particular for samples annealed at 500 °C for 5 minutes. These class of samples have been chosen, as explained later in the text, as probe transducers for monitoring refractive index changes at the metal interface with the aim to compare their functional abilities with the related MO signals.

### Magneto-Optical characterization

**Figure 3:**
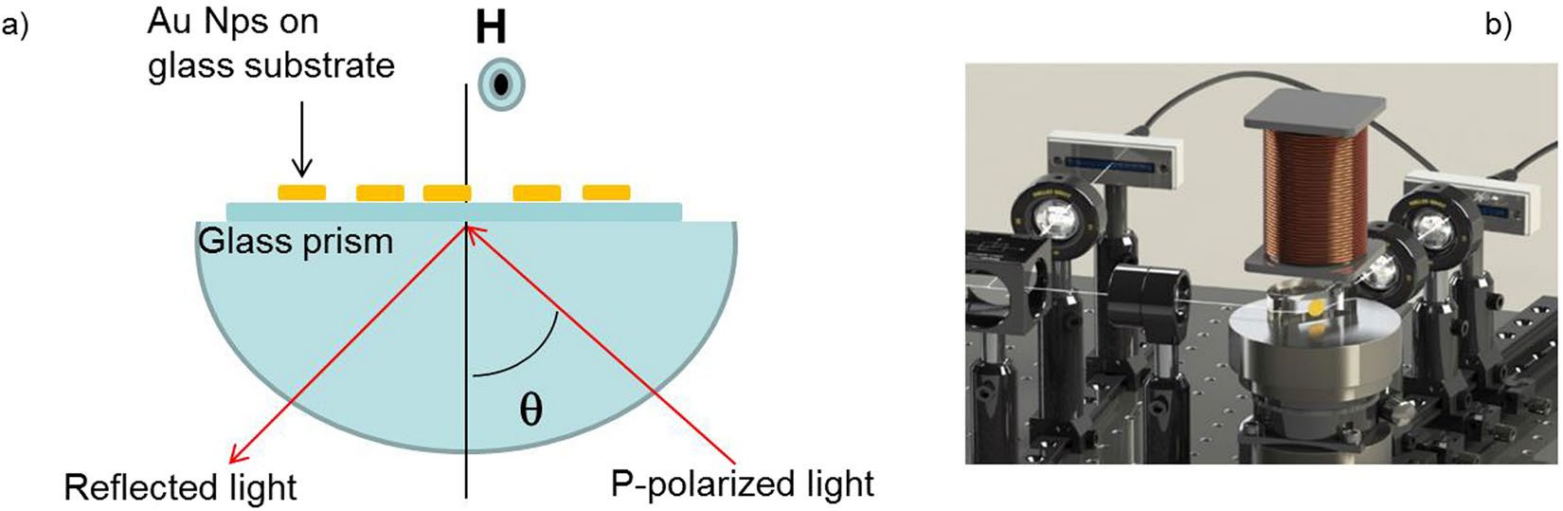
(**a**) Scheme and (**b**) experimental set-up for plasmonic and MO characterization in Kretschmann configuration using wavelength modulation.

An homemade experimental setup for optical and MO measurements was realized as reported in Fig. 3. In particular, MO signal is obtained by the external modulation of magnetic field in transversal configuration.

**Figure 4:**
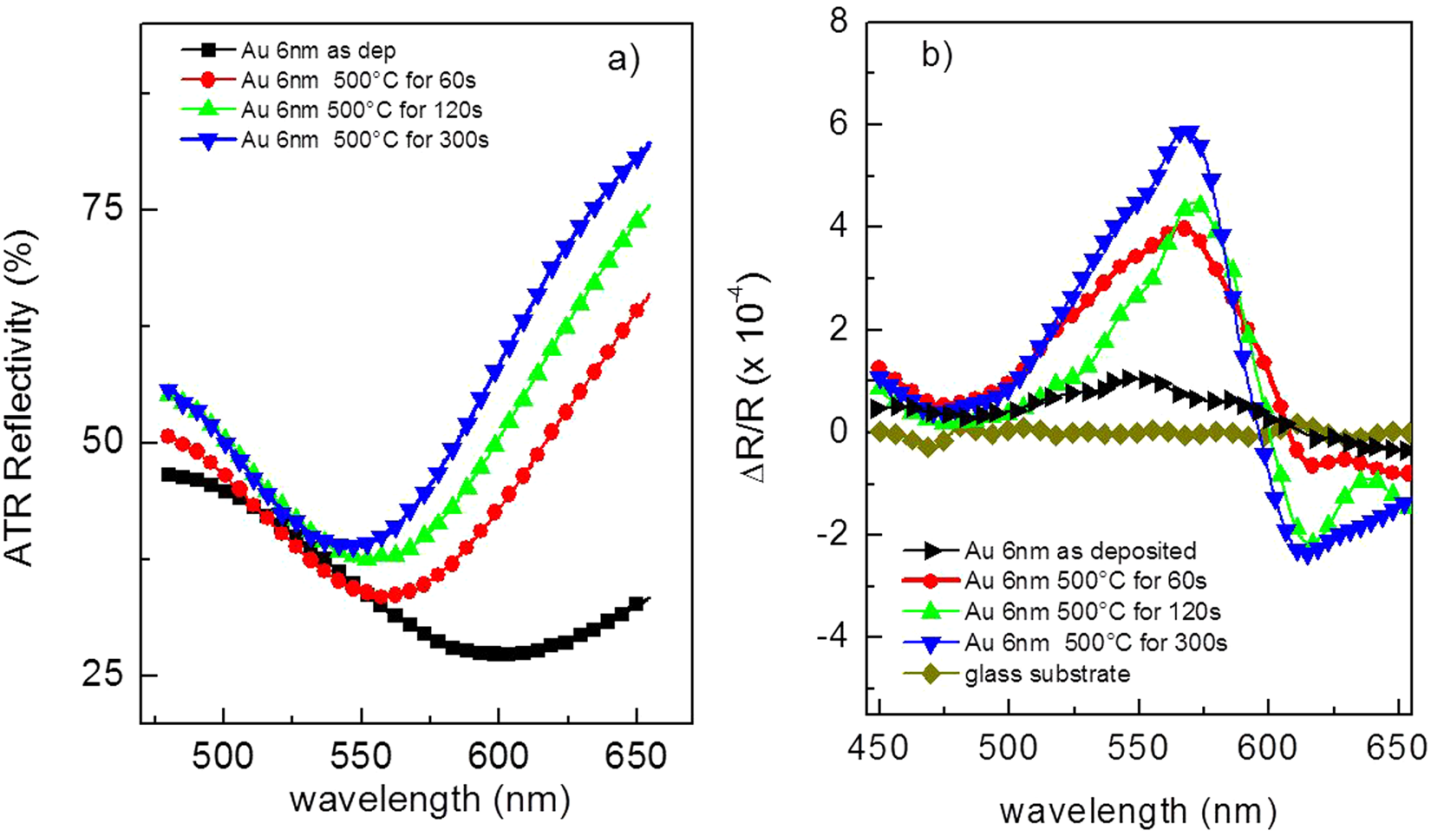
(**a**) Reflectance and (**b**) TMOKE curves recorded both in Kretschmann configuration in the same experimental setup and experimental conditions for the metal nanostructures obtained at increasing annealing times.

In Fig. 4a, we reported the reflectance spectra recorded in Kretschman configuration, using p-polarized light impinging at 55° with respect to the surface normal, in the 450–650 nm spectral range. Excitation of the LSP (seen as a minimum in the spectrum) at this incidence angle takes place at a wavelength of ca. 570 nm. In Fig. 4b, the corresponding TMOKE spectra upon application of an external modulated magnetic field, as detailed above, are reported in the same spectral range for the same set of samples. The MO signal of the bare glass substrate is reported as well, demonstrating a null contribution to the MO activity of the glass supported-metal nanostructures. When the resonance condition of gold nano-particles is satisfied, a s-like feature in the MO spectra can be clearly seen. This effect is more pronounced for samples reporting the best coupling between light and plasmon oscillations, namely those samples obtained at higher annealing time. The relation between the optimum plasmonic excitation condition and the intensity and shape in the MO curve confirms the coupling between plasmonic and MO properties of the nanostructures.

In the next section we will use the sharp feature in the s-like structure to evaluate its performance as refractive index sensing transducer. To this purpose, samples annealed at 500 °C during 300 s have been chosen as functional transducer probes, as explained in detail in the following. For the sake of completeness, an hysteresis loop has been reported in Fig. S2, while MO signal without TIR excitation is reported in Fig. S3. Related absorption spectrum obtained at the same incident angle of the TIR-excited setup is reported in Fig. 2b.

### Numerical Results

**Figure 5:**
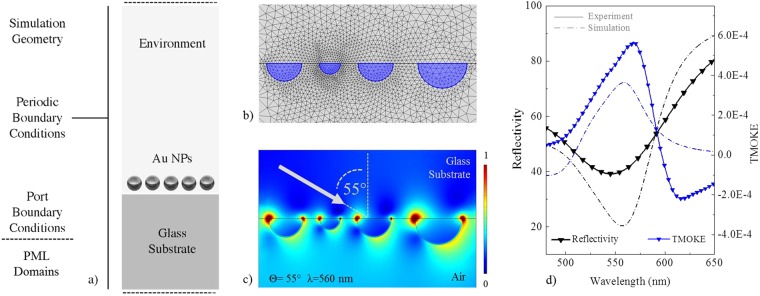
(**a**) Simulation geometry for an ordered array of Au NPs deposited on a glass substrate; (**b**) Free triangular mesh distribution used for the discretization of the simulation domain with a local refinement on plasmonic NPs; (**c**) A 2D plot of the Electric field Norm distribution calculated at the resonance frequency of the system for an incident angle of 55°. A great enhancement of the E field can be noticed near particles surface when the LSPR is activated; (**d**) Comparison of theoretical (dash dot line) and experimental (solid line) results obtained in Kretschmann configuration at an incident angle of 55° degree; in particular, the optical (black line) and MO (blue line) spectra of the analysed system have been compared.

To theoretically verify the existence of magneto-plasmonic coupling effects on nanostructured materials made of noble metals we will address the optical and MO response of planar distribution of gold hemi-spheres on glass substrates solving Maxwell’s equations within the Finite Element approach. In our case we have chosen the morphology of gold nano-islands to have hemispherical geometry. By using the results of SEM morphological analysis, we built four series of samples characterized by nano-hemispheres with three different diameters: 15, 25 and 35 nm. These average values have been calculated by considering the random distribution of real islands sizes obtained by morphological characterization (Fig. 5).

The simulation domain, partially illustrated in Fig. 5a, is composed by four layers with optimized thicknesses, and represents the unit cell of the analysed periodic array. In order to avoid possible multiple reflections inside the simulation domain, Perfectly Matched Layers (PMLs) conditions have been set for the outer regions of the model. Port boundary conditions automatically determine the reflection and transmission coefficients calculating the scattering parameters (S-parameters). Central domains represent the glass substrate and the environment and are characterized by wavelength dependent optical properties.

The guiding principle in the FEM Analysis is a local approximation of the solution. This approximation is closely associated with a fine discretization of the computational domain into small subdomains called elements or mesh. In the model a free triangular mesh has been used for the environment, the substrate and the scattered domains with a local refinement on the particle geometry (Fig. 5b). A mapped mesh with a five layers distribution, has been adopted for the PML domains (data not shown).

Floquet periodic boundary conditions are used on the sides of the unit cell to simulate an infinite array of NPs with inter-particles distances of few tens of nanometers. The incident electromagnetic wave is excited from the port on the top of the model and is linearly polarized in the incident plane.

A parametric sweep in the frequency domain was performed in order to calculate the total EM field distribution and the optical and MO response of the system, as function of the light frequency. As can be noticed in Fig. 5c, a great enhancement of the electromagnetic field arises in proximity of the nanostructures when resonance conditions are satisfied. The resonantly enhanced electron oscillations inside each nano-hemisphere generate dipolar fields on the outside of the particles. As we previously pointed out, these collective plasma oscillations are responsible for the enhanced absorption and scattering of light, as well as the strong confinement of EM fields in proximity of the NP surface (hot-spots). The optical response of the nanostructures, reported in Fig. 5d, exhibits the expected well-pronounced minimum in their wavelength-dependent reflectivity associated with the excitation of LSPRs. The wavelength yielding the strongest coupling, i.e. the *resonance wavelength*, was found to be around 560 nm, which is very close to the experimental value. The small difference between simulated and experimental reflectivity spectra may be explained by the slight difference in sizes distribution between real samples and model that, accurate as it is, it inevitably cannot match perfectly the disordered real nature of size and shape of experimentally analysed nanostructures. The coupling between MO and plasmonic effects can be reasonably understood considering the off-diagonal (OD) elements of the involved materials. In the TMOKE configuration, chosen for our experiments, the ε_xz_ and ε_zx_ components are the ones responsible for the MO activity of the system. Since the response of materials to alternating fields is characterized by a complex permittivity ε, each element of this tensor becomes a *frequency dependent complex function*, which can be separated in its real and imaginary parts as follow:

$${\varepsilon }_{xx}={\varepsilon }_{yy}=\varepsilon ={\varepsilon }_{1}(\lambda )-i{\varepsilon }_{2}(\lambda )$$

$${\varepsilon }_{xz}=-\,{\varepsilon }_{zx}=i\varepsilon ^{\prime} ={\varepsilon ^{\prime} }_{1}(\lambda )-i{\varepsilon ^{\prime} }_{2}(\lambda )$$

Therefore, in our numerical calculations, both the optical and MO properties of the gold nanostructures and the glass substrate have been described by introducing the real and the imaginary parts of the non-zero elements of ε. In plasmonic materials like noble metals, the off-diagonal elements are generally several orders of magnitude smaller with respect to ferromagnetic materials, therefore, in our calculations, the effect of an external magnetic field applied in TMOKE configuration was simulated introducing an off-diagonal element ε_xz_ = 10^−4^ + i10^−3^ (valid for Au at 700 nm and 1 T)^12–15^. In this case the LSP activation was performed using the wavelength modulation technique, simulating an incident light beam with a fixed incident angle (55° degrees in Kretschmann configuration) and multiple incident wavelengths (450–650 nm).

When the resonance condition of gold nano-particles is satisfied, an evident enhancement of the MO activity is predicted to exist. An increased MO activity appears at the same spectral position of the reflectivity reduction associated to the LSPR excitation, suggesting an intimate connection between plasmonic and MO effects (Fig. 5d). In addition, both reflectivity and TMOKE spectra exhibit similar redshifts when the incident angle of the incoming radiation increases. Although the amplitude of the theoretical MO signal is slightly smaller than the experimental values, our numerical results yield very similar features to those experimentally found for a random distribution of gold nanospheres.

The above result finds an experimental confirmation in the mathematical description reported in Lodewijks *et al*.^4^ work for magnetic nanostructures deposited randomly on glass substrates. The additional point here is the demonstration of such MO effects in Au materials when realized on the nanoscale. Analogue result was achieved in Polar configuration by Sepulveda *et al*.^15^. Unlike them, in our work a demonstration of MO properties in a TMOKE configuration with LSPR excited in TIR gives the perspective of an application in a potential sensing device.

In addition to the Lodewijks achievements, Maccaferri *et al*.^16^ investigated also the effect of the insulating substrate on the MO activity of magnetic nanostructures demonstrating that it affects the phase of non-magnetic Fresnel coefficient contributing in the changes of MO parameters recorded in L-MOKE and P-MOKE configurations. In order to identify correctly the origin of the MO effects, the possible MO properties of the glass substrate have been considered in our calculations too. To this purpose, the previously reported results, have been compared to those obtained from nanoparticles deposited on a glass slide with nonzero off-diagonal elements of the dielectric tensor ε (Fig. S4). The results lead to the conclusion that no significant contribution from glass is noticeable.

### Physical aspects behind the MO activity of metal nanostructures

It is important to observe that according to the MIE theory of single particles^17,18^, when the size of the nanoparticles is reduced, the normal electromagnetic modes are described by the relation

1 $$\varepsilon (\omega )=-\,\frac{l+1}{l}{\varepsilon }_{d}\,$$

consequently different polarizations can be induced to switch-on electrical dipoles that, in the case of *l* = 1 give rise to a LSPR of single particles $\varepsilon (\omega )=-\,2{\varepsilon }_{d}$, with polarization vector ***P*** given by the relation^19^:

2 $${\boldsymbol{P}}=\frac{3}{4\pi }[\frac{\varepsilon (\omega )-{\varepsilon }_{d})}{\varepsilon (\omega )+2{\varepsilon }_{d}}]{{\boldsymbol{E}}}_{{\boldsymbol{ext}}}$$

that produces an oscillating surface charge onto the nanoparticles. A Maxwell-Garnett Effective Medium Approximation in which the effective dielectric constant *ε*_*eff*_ is introduced can be used to describe assemblies of randomly distributed nanoparticles^18^. For a system containing metal nanoparticles in a host material the *ε*_*eff*_ value is given by:

3 $${\varepsilon }_{eff}(\omega )={\varepsilon }_{d}1+3f\frac{\varepsilon (\omega )-{\varepsilon }_{d}}{\varepsilon (\omega )(1-f)+{\varepsilon }_{d}(2+f)}$$

where *f* is the filling factor defined as the volume fraction occupied by the nanoparticles and the resonance condition takes place at

4 $$\varepsilon (\omega )=-\,\frac{2+f}{1-f}{\varepsilon }_{d}$$

At low density of nanoparticles we have *f* ≅ 0 and the condition of single particle is obtained. If *f *≠ 0, the resonance frequency shifts to a lower value because of dipole-dipole interaction. The simplest nanoparticle aggregate consists in a couple of nanoparticles that can be longitudinally or transversally excited by polarized light.

**Figure 6:**
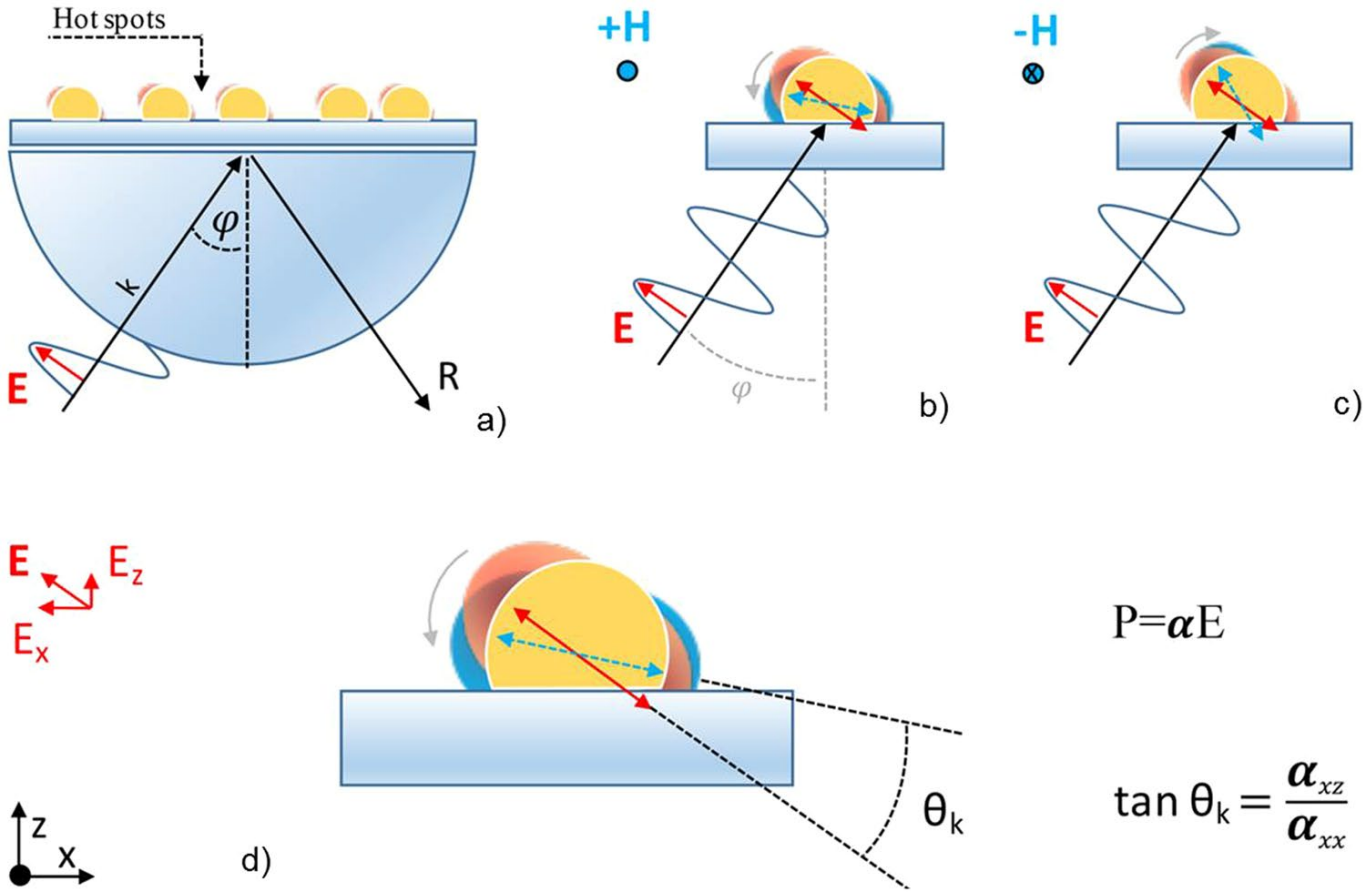
(**a**) Hot spots generate by polarized incident light onto the nanostructured material. Schematic of the MO effect induced by the Lorentz force in metal nanoparticle, (**b**) before and (**c**) after the application of an oscillating magnetic field in transversal configuration. (**d**) Schematic representation of the torque suffered by the oscillating electrical dipoles due to the magnetic field application causing the switch-off of the hot spots in the plane of the substrate.

In real samples, like in randomly glass supported-Au nanostructures studied in this work, the nanoparticles exist as aggregates or high density distribution, and the presence of an incident p-polarized light products a random hot-spots distribution. From an experimental point of view, the hot-spots generated by the p-polarized incident light, can be modified and even switched-off if the polarization of the incident light is out of the substrate plane. Obviously, in Kretschmann configuration the incident light is not at normal incidence then, a component of the electric field perpendicular to the plane of the sample can be observed. This component can be modified by changing the incident angle of the light or, at the same incident angle, by the application of a magnetic field in the plane of the nanostructured sample and perpendicular to the incident plane of light. We demonstrate here that a low intensity modulated magnetic field can be suitable to torque the oscillating electrical dipoles (Fig. 6) of noble metal NPs, generating a detectable MO effect in T-MOKE configuration.

Any movement of charge q with speed **v** in presence of a magnetic field **H** gives rise to a Lorentz force *F* = *q****v*** × ***H*** If one considers the activation of oscillating dipoles by p-polarised light of suitable wavelength, the presence of an external magnetic field will generate a torque of the electric dipoles according to the equation ${\boldsymbol{F}}=(d{\boldsymbol{P}}/dt)\times {\boldsymbol{H}}$ where **P** = α**E** with α polarizability of the material and E amplitude of the incident polarized light beam^20^. Generally, in the presence of a magnetic field, the polarizability $\tilde{\alpha }$ is a tensor described by the relation^21^:

5 $$\mathop{\alpha }\limits^{ \sim }=\frac{4\pi {a}^{3}(\mathop{\varepsilon }\limits^{ \sim }-{\varepsilon }_{d}\mathop{1}\limits^{ \sim })}{(\mathop{\varepsilon }\limits^{ \sim }+2{\varepsilon }_{d}\mathop{1}\limits^{ \sim })}\,$$

Where $\tilde{1}$ is the 3 × 3 identify matrix, $\tilde{\varepsilon }$ is the dielectric tensor of metal, *ε*_*d*_ is the dielectric constant of the external medium, *a* is the size of the nanoparticle. If the **H** vector is in the plane of the sample and perpendicular to the incidence plane of the light (transversal configuration) the dielectric matrix reports only the two not diagonal elements ε_xz_ and ε_zx_. In this condition, substituting the values to the Eq. 5 we obtain for the components of interest in T-MOKE configuration:

6 $${\alpha }_{xz}\cong -\,{\varepsilon }_{xz}(\varepsilon +2{\varepsilon }_{d})(2\varepsilon +{\varepsilon }_{d})$$

and

7 $${\alpha }_{xx}\cong 4\pi {a}^{3}{\varepsilon }_{d}({\varepsilon }^{2}-4{\varepsilon }_{d}^{2})$$

Consequently, the torque suffered by the ***P*** vector due to the presence of an external magnetic field in TMOKE configuration is quantified by the *θ*_*k*_ angle (Fig. 2a,b) given by:

8 $${\theta }_{k}=arctg\,\frac{{\alpha }_{xz}}{{\alpha }_{xx}}=arctg\frac{-\,{\varepsilon }_{xz}(\varepsilon +2{\varepsilon }_{d})}{4\pi {a}^{3}{\varepsilon }_{d}(\varepsilon -2{\varepsilon }_{d})}$$

here we put in evidence that $\varepsilon \approx \,{\varepsilon }_{xx}\approx {\varepsilon }_{yy}\approx {\varepsilon }_{zz}\approx ({\varepsilon }_{\infty }-\varepsilon (\omega ))$with $\varepsilon (\omega )=\frac{{\omega }_{p}^{2}}{\omega (\omega +i\gamma )}\,\,$where ω_p_ is the plasma frequency of the electron’s gas, γ is the electronic relaxation constant and ${\varepsilon }_{\infty }$ is the high frequency dielectric constant.

In Fig. 6 a generic scheme of the hot-spots generated in resonance condition and the effect of the application of a magnetic field in transversal configuration is reported.

### Functional characterization

As a proof of concept, the recorded MO signal of gold nanostructures has been tested as a new probe for refractometric sensing tests.

Investigation on sensing performances of MO signals is not a trivial question, especially if comparison with bare optical signals is required. Different approaches can be found in the huge plasmonic sensing literature; in most of the wavelength-modulation schemes, the quantity often chosen as a measure of the sensing performance is linked to spectral positions shifts upon bulk refractive index changes of the dielectric medium. Analogously, when the transducer signal is a wavelength-modulated MO signal, different authors make a similar choice. Caballero and Diaz-Valencia monitored as functional parameters the bulk refractive index sensitivity (RIS) intended as the spectral shift in MO Fano-like transducing signal with respect refractive index changes in the investigated medium^22^. In order to take into account that sensing signal accuracy depends on the spectral line width, a further figure of merit (FoM) is considered by dividing the bulk sensitivity S by the width of the Fano-like feature. A different approach has been proposed by Jeong *et al*.^23^; they performed Circular Dichroism-based measurements on chiral particles plotting the reciprocal of the optical feature. This choice allowed them to get an intrinsically sharp representation of the spectral signal and consequently to maximize FOM parameter. None of the above authors have reported a comparison of the sensing performances with the corresponding bare optical sensing signal except with literature. Some efforts in this sense have been reported in Verre *et al*.^24^ where LSPR sensing based on polarization conversion in anisotropic gold nanostructures is demonstrated experimentally. Transient measurements were performed by changing the bulk RI and monitoring both the modulated rotation signal and the standard transient transmission on the same sample demonstrating that the last one is too noisy to follow small refractive index variation. Analogously, Vavassori *et al*. investigated magneto-optically induced LSP resonances in magnetoplasmonic nanoantennas by monitoring the polarization ellipticity variation of the transmitted light, induced by the application of an external magnetic field. Comparison with bare plasmonic-based sensors performance is achieved by monitoring the null-point wavelength in the polarization ellipticity variation and in particular the spectral position of the reciprocal of this MO signal thus resulting in greatly enhanced values for the FoM^25^. They found even highly enhanced FoM values when periodic array of Ni/SiO2/Au dimer nanodisks were realized. The sharp spectral features obtained, due to combined effects of near-field interactions between the Ni and Au disks and far-field diffractive coupling between the dimers of the array, gave rise to improved sensing performance^26^.

In all above examples, the existence of Fano-like curve are the key point for successful sensing purposes. In fact, the presence of a ferromagnetic material, either alone or in combination with noble metals, dictates the MO response resulting in very deep transducing signals allowing enormous gain in the spectral accuracy. In plasmonic-based sensors, monitoring spectral position variations upon refractive index changes becomes a natural choice in particular for those nanostructures where scattering is a major component of the total extinction^27^. In the present work, intensity changes, either related to the optical or MO signal, has been chosen to quantify refractive index changes. This choice is in part dictated by the adopted TIR configuration for the excitation of LSPR resonances allowing also a dynamic measurement of the signals, but also to the fact the optical information is basically dominated by absorption.

We performed a series of measurements in which the gold nanostructures deposited onto glass substrate are located in a suitable chamber for test in a flow-over arrangement in Kretschmann configuration. Liquid solutions with a known refractive index were injected in the test chamber to register the corresponding calibration curves. The ability of the investigated transducers to act as a refractive index sensor was investigated at a wavelength of 590 nm by monitoring the intensity of both LSPR and MOLSPR after their immersion in solutions with different refractive indices (RI): air (*n* = 1), pure ethanol (*n* = 1.36), water (*n* = 1.33) and in a water/ethanol solutions at increasing concentrations. Among the grown nanostructures, samples annealed for 5 minutes has been chosen for the functional characterization, as they exhibit the narrow excitation so that it can be sensitively modulated by an external magnetic field.The wavelength of 590 nm was selected by considering not only the region of the maximum slope of the LSPR curve but also the region in which the slope of the ΔR/R signal is maximum.

Once determined the sensing parameter to be monitored, the comparison of optical and MO functional parameters cannot be carried out without considering the different nature of the transducing signals. To this purpose, following the route proposed by Sepulveda *et al*.^5^, monitoring of each sensing signal is carried out with a normalization coefficient taking into account the different the signal-to-noise ratio of the experimental measurements.

**Figure 7:**
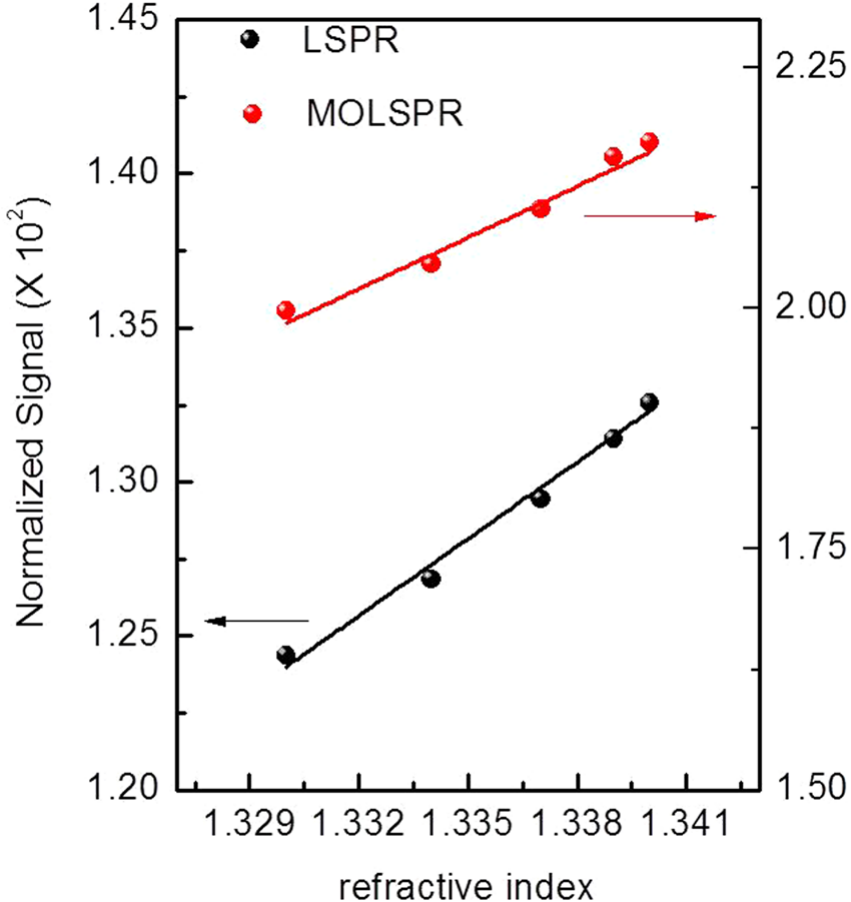
Calibration curves obtained in LSPR and MOLPR configuration relative to bulk refractive index changes at the metal/dielectric interfaces.

Comparison of the calibration curves relative to the two sensing platforms is reported in Fig. 7; here the sensor response is represented by the sensor signal normalized by a minimum detectable signal intended as five times the root-mean-square deviation of the experimental signal acquired during 300 s when 1 point/s is acquired.

Linear regression of calibration points allows extracting information on the sensitivity parameters of both sensor signals: an improvement in the sensing performance of the MO-LSPR transducer with respect to LSPR is demonstrated by slopes of the linear fit resulting in a sensitivity of 1.8 × 10^3^ and 8.3 × 10^2^ N.I.U.(normalized intensity unit) per RIU, respectively.

RI limit of detection parameter can be considered as a the most reliable literature term of comparison. By exploiting the above calculation of sensitivity, which takes into account the different nature of the two recorded sensing signals, the obtained RI resolution are: 5.6 × 10^−4^ RIU and 1.2 × 10^−3^ RIU for MO-LSPR and LSPR sensing probe respectively. The results obtained for the bare plasmonic transducers are basically aligned with those reported in the literature for similar metal nanostructures^21,28–31^ while an increase of one order of magnitude in the sensing performance of the MO-LSPR based sensor. Even if exploratory, the result is of great impact and high promising for state-of the- art sensors and biosensor. There is still plenty of room for improving the results reported here by modifying, for instance, the growth parameters, distance and shapes of metal nanostructures, or by a different choice of the metal.

## Conclusions

In this paper glass supported- random distribution of Au nanoparticles were realized and characterized by a morphological and optical point of view. Optical absorption spectra of sample exposed to increasing annealing treatments represents allowed monitoring the reproducibility of the preparation methodology of active nanostructured optical transducers. Experimental as well as theoretical demonstration of their MO behaviour, obtained for the first time in transversal configuration (TMOKE), have been reported. A FEM method was used to calculate the optical and MO spectra, investigating the interplay between plasmonics and MO effects, taking their specific morphology and distribution onto the glass substrate into account. The developed numerical models provided invaluable physical insight of our nanostructured systems and have been also exploited for the validation of our experimental findings. The investigated effect has been described by considering the presence of Lorentz Forces acting on the electrical dipoles generated in resonance (LSPR) condition. The Kretschmann configuration adopted in our experiments supported the potential application of the TMOKE signal in refractometric sensing application. An increase of one order of magnitude in the sensing performance of the MO-LSPR based sensor with respect to the related LSPR sensing transducer is recorded.

## Methods

### Deposition of gold nanostructures

The main routes for the preparation of the Au nanostructures are based on the use of photons (multiple beam interference lithography)^32^, self-assembling^33,34^, colloidal lithography^35–37^, electron beam lithography (EBL)^38,39^, or focused ion beam (FIB)^40,41^. Some of them, even though ensuring high control of spatial and size regularity, suffer of high costs and are rather time consuming; therefore, if these aspects are not strictly required, the choice of a low cost and fast (or time-saving) fabrication techniques should be considered.

In our choice, gold nanoparticles were prepared by a physical methodology consisting in electron beam evaporation of a thin Au film onto corning glass substrates (10 × 10 mm^2^ an 1 mm in thickness) followed by a rapid thermal annealing treatment. The substrates were first degreased by rinsing in boiling acetone for 20 minutes, cleaned in iso-propanol vapors for 1 h and finally dried under pure nitrogen (N_2_) flow. The substrates were then loaded in a Joule evaporator UHV chamber for the subsequent deposition of the Au thin film. A film thickness of 6 nm was obtained at the evaporation rate of about 0.02 nm/s, as determined by a quartz crystal oscillator thickness monitor. After evaporation, the samples were loaded into the quartz chamber of a horizontal tubular resistance furnace for annealing under nitrogen atmosphere. Annealing experiments were performed for different annealing time of 30, 60 and 120 seconds at 500 °C under 200 cm^3^/min pure N_2_ flow. This thermal treatment allows the Au thin film to coarse into a dense and uniform array of metal nanoparticles.

### Morphological and optical characterization

A full morphological, optical and magneto-optical (in T-MOKE configuration) characterization of the resulting Au nanostructures prepared onto solid transparent substrates has been performed.

The morphology and size of the Au nanoparticles were characterized by Scanning Electron Microscopy (SEM), by using a Zeiss NVISION 40 dual-beam Focused Ion Beam machine, equipped with a high resolution SEM Gemini column.

The optical absorption of the Au nanoparticle deposited onto glass substrates was characterized by a Cary500 UV-visible spectrophotometer. Investigation of the effect of thermal annealing on the nanoparticle optical properties was performed by measuring the absorption spectra in the UV-VIS spectral range for different samples at increasing annealing time.

### Magneto-Optical Localized Surface Plasmon Resonance (MOLSPR) experimental set-up

The homemade setup for MO measurements was realized in the Plasmonics and Nanophotonics Advanced Laboratory at the CNR Institute for Microelectronics and Microsystems (IMM-CNR), in Lecce (Fig. 1). The experimental setup with the relative signal data processing is designed in such a way that either optical and MO excitation is achievable on the same sample. In particular, MO signal in transversal configuration is obtained by the external modulation of magnetic field, namely with magnetic field applied parallel to the sample plane and perpendicular to the light incidence plane. Illumination is achieved by a polychromatic 150 Xe light source (Lot-Oriel LSB 520) with emission in the 300–900 nm spectral range. A monochromator is placed between the source of light and the sample, allowing the analysis of plasmonic and MO effect as a function of wavelength. Then, the cross section of the light beam was reduced by an achromatic doublet of lenses of proper focal lengths, which allows focusing the radiation on the sample in a small spot (1 mm × 3 mm). Also the required p-polarized light (electric field parallel to the plane of incidence) was achieved introducing a linear polarizer on the optical bench. Then, the reflected beam coming from the prism/sample structure reaches the surface of a silicon photodiode. The plasmonic transducer is coupled to a commercial glass prism (BK7), transparent within the wavelength range of interest, with an average refractive index equal to n = 1.516. The prism/sample combination is placed on a rotating structure θ-2θ, which allows the user to manually control the angular position of the sample (resolution of 0.01°). Moreover, the sensor surface is placed into a test chamber connected to the fluid control system. This experimental set-up allows analyzing the optical and MO responses of the sample in a controlled atmosphere. A dedicated software developed with LabView handles the experimental data acquisition and processing. Through a simple graphical interface, the program allows controlling the wavelength of the incident light and obtaining a real time reading of the generated photocurrents. An external magnetic field of 500 Oe intensity oscillating with an 20 Hz frequency was used for TMOKE experiments and lock-in amplification technique was used for recording the modulated reflectance signal. The DC component of the signal is processed by the electronic card in order to record the intensity of the reflected light for the determination of the bare reflectivity signal. This system is directly linked to the user PC using a National Instruments PCI GPIB parallel interface, and allows knowing in real time the intensity of the radiation that reaches the prism-sample system.

### Numerical methods

In order to shed light to our experimental findings and perform an in-depth investigation of the interaction between LSPR and MO effects on metal nano-particles, we carried out a complementary theoretical analysis based on numerical simulations. In particular, the Finite Element Method (FEM) was used to help understanding the optical and MO properties of gold nanoparticles deposited on glass substrate.

Although not a physically realistic configuration, the infinite-array model provides a good approximation to the performance of a large finite array of particles. The Radio Frequency Module of COMSOL Multiphysics software (www.comsol.com) was used to calculate the optical transmission and reflection coefficients of these structures, when are illuminated by a linearly polarized plane wave propagating along a defined direction.

The optical properties of the system were investigated in a particular frequency range, corresponding to a free space wavelength from 450 nm to 650 nm. At these frequencies, nanostructured gold can be modelled as having a complex valued dielectric constant, with real and imaginary components. The complicated dispersion properties of metals at optical frequencies poses a challenge in modelling of plasmonic devices. However, the chosen method allows using directly experimental data for the frequency-dependent dielectric function of the involved materials, including both the real and imaginary parts, with no further approximation. In the model, these components are described by an interpolated version of the often-used experimental data from Johnson and Christy^42^.

## Electronic supplementary material

Supplementary Information
