# Supplementary material for: Magneto-Optical properties of noble-metal nanostructures: functional nanomaterials for bio sensing
Source: Sci Rep. 2018 Aug 23;8:12640. doi: 10.1038/s41598-018-30862-3 (PMC6107575; doi:10.1038/s41598-018-30862-3)
Supplement: Supplementary file 1 — Supplementary Information [file 41598_2018_30862_MOESM1_ESM.docx]

**Supplementary information**

**Magneto-Optical properties of noble-metal nanostructures:**

**functional nanomaterials for bio sensing**

M.G. Manera^1^, A. Colombelli^1^, A. Taurino^1^, A. Garcia Martin^2^, R. Rella^1^

^1^ Istituto per la Microelettronica e i Microsistemi IMM sezione di Lecce, Via per Arnesano,73100 Lecce (Italy) –

^2^ IMM - Instituto de Microelectrónica de Madrid (CNM-CSIC), Isaac Newton 8, E-28760 Tres Cantos, Madrid, Spain

Correspondence and requests for materials should be addressed to Roberto Rella

(email: roberto.rella@cnr.it)

***
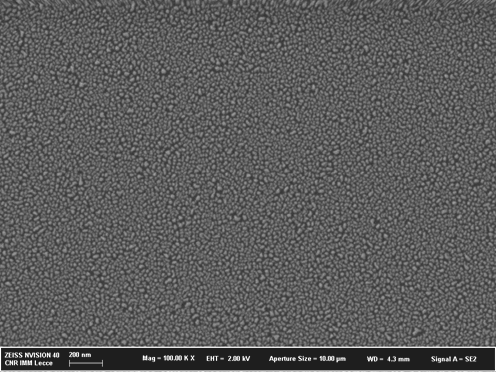
***

Figure S1. Morphology of the nanostructured gold nanoparticles obtained by SEM relative to the as deposited layer

Figure S2. Kerr loops in Krestchmann configuration of Au nanostructures deposited on glass substrated (sample annealed for 5minutes) measured at λ=570nm in transversal configuration


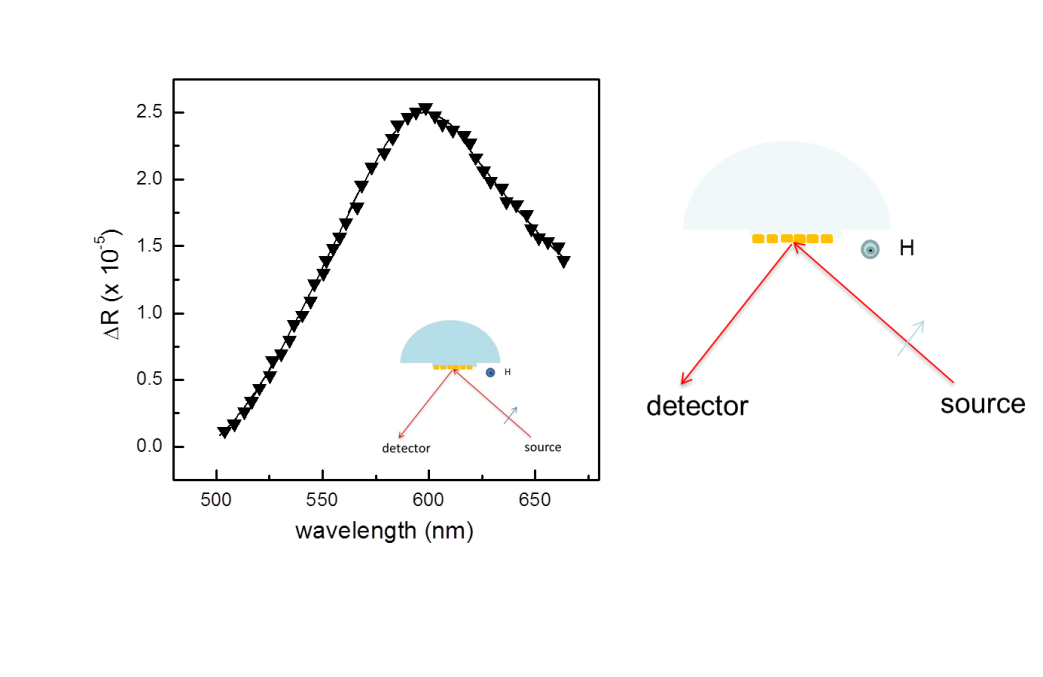


Figure S3. Morphology of the nanostructured gold nanoparticles obtained by SEM relative to the as deposited layer


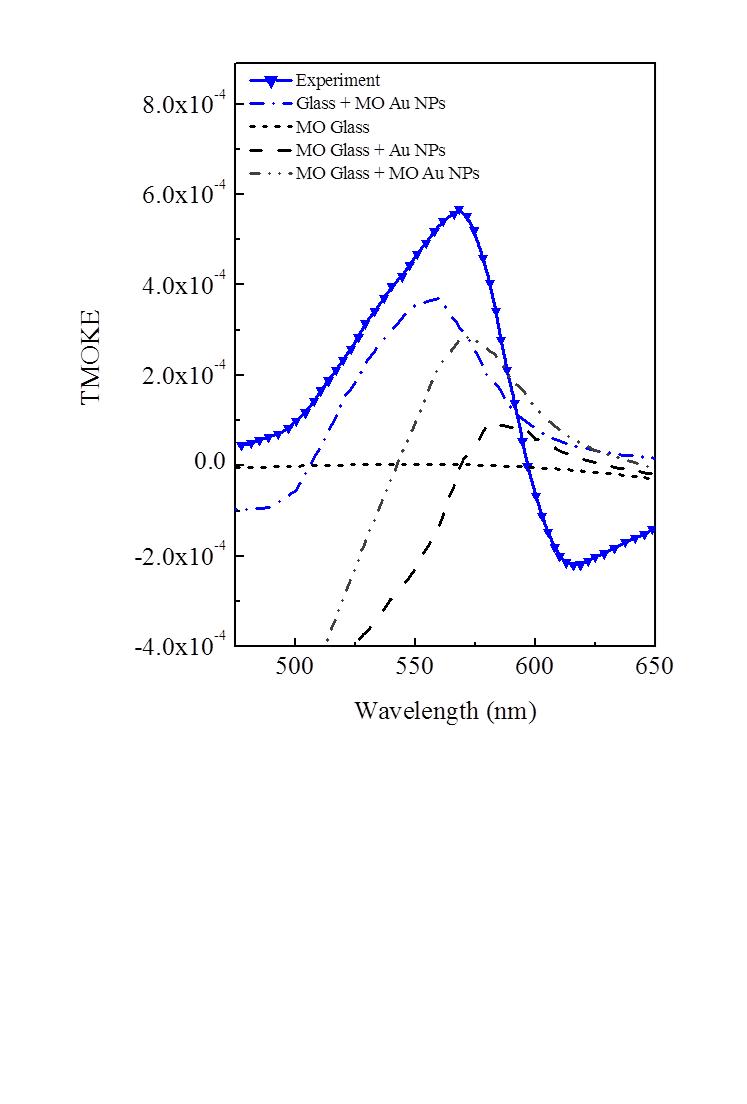


Figure S4. Black curves represents MO curves calculated by considering the bare contribution of non-null off diagonal components of glass substrate (MO glass); their combination with null off -diagonal elements of Au nanostructures (MO glass+Au NPs; their combination with non- null off -diagonal elements of Au nanostructures (MO glass+ MO Au NPs. Blue curves represents MO curves calculated by considering only the non-diagonal components of the Au nanostructures (dashed lines); comparison with experimental results is reported as well (triangles+line)
